# Interaction between the Number of Chemotherapy Cycles and Brachytherapy Dose/Volume Parameters in Locally Advanced Cervical Cancer Patients

**DOI:** 10.3390/jcm9061653

**Published:** 2020-06-01

**Authors:** Alexandre Escande, Mohamed Khettab, Sophie Bockel, Isabelle Dumas, Antoine Schernberg, Sebastien Gouy, Philippe Morice, Patricia Pautier, Eric Deutsch, Christine Haie-Meder, Cyrus Chargari

**Affiliations:** 1 Brachytherapy Unit, Gustave Roussy Cancer Campus, F-94800 Villejuif, France; sophie.bockel@gustaveroussy.fr (S.B.); isabelle.dumas@gustaveroussy.fr (I.D.); antoine.schernberg@gustaveroussy.fr (A.S.); Christine-haiemeder@gustaveroussy.fr (C.H.-M.); cyrus.chargari@gustaveroussy.fr (C.C.); 2 Radiotherapy Department, Gustave Roussy, F-94800 Villejuif, France; mohamed.khettab@gustaveroussy.fr (M.K.); eric-deutsch@gustaveroussy.fr (E.D.); 3 Academic Radiotherapy Department, Oscar Lambret Comprehensive Cancer Center, F-59800 Lille, France; 4 Surgery Department, Gustave Roussy Cancer Campus, F-94800 Villejuif, France; sebastien.gouy@gustaveroussy.fr (S.G.); philippe.morice@gustaveroussy.fr (P.M.); 5 Medical Oncology, Gustave Roussy Cancer Campus, F-94800 Villejuif, France; patricia.pautier@gustaveroussy.fr; 6 Biological Effects of Radiation, Institut de Recherche Biomédicale des Armées, F-91220 Bretigny-sur-Orge, France; 7 French Military Health Academy, Ecole du Val-de-Grâce, F-75005 Paris, France

**Keywords:** locally advanced cervical cancer, chemoradiation, chemotherapy, image-guided adaptive brachytherapy

## Abstract

Scarce data exist on concurrent chemotherapy in locally advanced cervical cancer (LACC) patients (pts) treated with image-guided adaptive brachytherapy (IGABT). We examined the effect of a number of chemotherapy cycles and their interaction with brachytherapy dose/volume parameters. Clinical records of 209 consecutive pts treated for a LACC were reviewed. Pts received CRT concurrently with cisplatin 40 mg/m² or carboplatin AUC2. An additional cycle could have been delivered during the pulse-dose rate (PDR)-IGABT. The impact of a number of chemotherapy cycles on outcome was examined, as well as the interactions with dose volume parameters. The number of cycles was four in 55 (26.3%) pts, five in 154 (73.7%) including 101 receiving the fifth cycle during IGABT. Median follow-up was 5.5 years. Pts receiving five cycles had a better outcome on all survival endpoints, including three year local control rate (93.9% vs. 77.2%; *p* < 0.05). In the subgroup, only pts with tumor FIGO (Fédération Internationale de Gynécologie Obstétrique) stage ≤IIB or with CTV_HR_ > 25 cm^3^ had a better outcome. Pts receiving four cycles with D_90_CTV_HR_ > 80Gy_EQD2_ had the same locoregional control–(LRC) as those receiving five cycles and achieving D_90_CTV_HR_ ≤ 80 Gy_EQD2_ (*p* = 0.75). An optimal propensity score matching the balance for the FIGO stage, CTV_HR_ volume and D_90_CTV_HR_ confirmed the effect, with the largest life expectancy benefit for locoregional failure-free survival (absolute gain: 1.5 years; *p* = 0.017). Long-term radiation-induced toxicity was not increased. Increasing the total number of cycles from 4 to 5 improved LFS, suggesting a place for systemic strategies aimed at in-field cooperation. Delivering an additional cycle at the time of brachytherapy did not increase morbidity and there permitted an increase in chemotherapy dose intensity.

## 1. Introduction

The benefit of concurrent chemoradiation (CCRT) in locally advanced cervical cancer (LACC) has been demonstrated for a long time, with large meta-analyses showing that the addition of chemotherapy to standard radiotherapy improved overall survival (OS) and progression-free survival (PFS), with absolute benefits of 10% and 13%, respectively. The benefit of concurrent CCRT was independent of platinum use, and relied on a dual effect on both local and distant failure, although the most significant benefit was in terms of local control, suggesting that there might be in-field cooperation [1,2,3,4,5,6,7,8].

More recently, the implementation of image-guided adaptive brachytherapy (IGABT) has drastically improved the outcome of patients with the advanced disease by increasing local control probability through dose escalation, with an increasing number of published studies suggesting that this benefit translates into a survival benefit [9,10,11,12,13]. The place of chemotherapy has been poorly documented in the era of IGABT, as meta-analyses on the benefit of chemotherapy were conducted without image guidance and without dose escalation. Although the important role of chemotherapy in the systemic control in high-risk patients treated with IGABT has been shown, the effect of chemotherapy on local control and interaction with dose/volume parameters in the era of modern irradiation techniques remains unknown [14].

In a large institutional series of patients homogeneously treated with CCRT followed by IGABT, we examined the effect of delivering an additional chemotherapy cycle concurrently with external irradiation and brachytherapy, and the extent of this effect according to brachytherapy dose/volume parameters.

## 2. Materials and Methods

### 2.1. Inclusion Criteria

Medical records of consecutive patients receiving curative intent in a single Comprehensive Cancer Center between 2004 and 2015 for histologically proven LACC were examined. Primary staging included magnetic resonance imaging (MRI) and 18-Fluorodeoxyglucose Positron Emission Tomography/Computed Tomography (PET/CT), completed with a primary para-aortic laparoscopic lymph node dissection in case PET/CT showed no extra-pelvic 18-FDG uptake. Staging exams could have been acquired in others hospitals but reviewed by a specialized radiologist team. Lymph node dissections were performed in the same hospital where treatment was delivered. Cancers were classified according to the Fédération Internationale de Gynécologie Obstétrique (FIGO) staging system of 2009.

The ability to deliver an appropriate number of chemotherapy cycles depends on various parameters that might bias analysis (e.g., age, general health status, infection of renal dysfunction related to tumour stage). In order to minimize biased analysis, only patients receiving 4 or 5 cycles of chemotherapy were analysed in order to have a more thorough analysis of the effect of receiving an additional chemotherapy cycle (4 vs. 5 cycles), either during external-beam radiation therapy (EBRT) or at time of brachytherapy.

Study design was conducted according to ethics rules and was approved by the institutional board for gynecological tumors.

### 2.2. Treatments

A pelvic external-beam radiation therapy (EBRT) was delivered in all patients, extended to para-aortic areas in case of para-aortic lymph node metastases. The radiotherapy dose was 45 Gy in 25 fractions of 1.8 Gy per fraction. Nodal boosts were delivered sequentially (in cases of 3D conformal radiotherapy) or concomitantly (in cases of intensity modulated radiotherapy), to deliver a total dose of 60 Gy to macroscopic lymph nodes metastases, taking brachytherapy contribution into account.

Treatment was followed by a utero-vaginal pulse-dose rate (PDR) brachytherapy boost guided by image and using the vaginal mold applicator technique, as previously reported, with the objective of having an overall treatment time (OTT) of less than 55 days [15]. Delineation of high risk clinical target volume (CTV_HR_), intermediate risk clinical target volume (CTV_IR_) and organs at risk (OAR) was done following GEC-ESTRO guidelines (*Groupe Européen de Curiethérapie*, European Society for Radiotherapy and Oncology). The treatment planning aim was to deliver at least 80 Gy to 90% of the CTV_HR_ (D_90_CTV_HR_) without exceeding OAR dose constraints. Treatment was delivered through continuous hourly pulses. Doses are reported in equivalent doses per 2 Gy fractions, using a linear quadratic model.

Concurrent chemotherapy was delivered: weekly cisplatin 40 mg/m² was administered by intravenous infusions from week 1 to week 5. In case of contraindication, such as a renal impairment, weekly carboplatin area under curve 2 was delivered. During CCRT, patients were seen weekly before the administration of chemotherapy for toxicity assessment. Chemotherapy doses were recalculated each week. Chemotherapy was not administered if there was a treatment break during radiotherapy or during the interval between EBRT and brachytherapy. Chemotherapy was suspended in case of neutropenia <1500/mm^3^ or platelets <100,000/mm^3^ and was resumed when polymorphonuclar neutrophils (PMN) were >1500/mm^3^ and platelets were >100,000/mm^3^. Leucocyte or granulocyte counts were not used. Chemotherapy was not interrupted in cases of anaemia as transfusion was performed when clinically indicated. Depending on blood cell count, an additional cycle could be delivered at the time of brachytherapy.

### 2.3. Follow-Up and Statistical Analysis

Follow-up was scheduled at 6–8 weeks then at every three months for two years, including a pelvic and abdominal MRI that was repeated every six months. Then, over the next three years, patients had a gynecological examination performed every 6 months and an MRI performed annually. Gynecological follow-up was scheduled annually. PET/CT were performed in case of a relapse or new symptoms. Factors associated with a tumor relapse were examined. All relapses were considered (not only first relapse) and classified as local (in the true pelvis), regional (pelvic and/or para-aortic nodal), locoregional (regional and/or local), or distant (metastasis). OS was calculated from the date of diagnosis. PFS, local relapse-free survival (LFS), locoregional relapse-free survival (LRFS) and metastasis-free survival (MFS) rates were estimated from the date of treatment initiation using the Kaplan–Meier method. The impact of chemotherapy on survival was assessed using Log a rank or Cox regression model after assumption assessment. Hazard ratios for event/death are reported. Differences in restricted mean survival time (RMST) were also reported and compared using maximal tau. Finally, propensity score analysis through the creation of a sample match set using optimal matching without replacement was used in survival analysis.

The effect of concurrent chemotherapy on cumulative long-term toxicities was assessed. Locoregional toxicities occurring after >6 months follow-up were scored according to the Common Terminology Criteria for Adverse Events version 3. Univariate analyses were performed after the exclusion of patients with a local relapse. Time to onset was defined from treatment initiation to the date of toxicity occurrence. Log rank tests were performed on Kaplan–Meier event-free period curves.

Statistical analyses were conducted using R studio, version 3.3.3 (RStudio, Inc., Boston, MA, USA). In multivariate analysis, only factors with *p*-value <0.1 were considered with a maximum of one factor per ten events. Associations between features and survivals were calculated using Spearman nullity test, Wilcoxon Mann–Whitney and Khi-2 tests depending on the type of factors. A *p*-value below 0.05 was considered as statistically significant.

The primary endpoint was to assess the impact of the number of chemotherapy cycles on survival and patterns of relapse.

Secondary endpoints included the description of interactions between the number of chemotherapy cycles and other prognostic factors, including dosimetric parameters, and also to examine the effect of the number of chemotherapy cycles on toxicity rate.

## 3. Results

### 3.1. Patients, Tumors and Treatments

A total of 209 patients fulfilled the inclusion criteria. The median age was 47.0 years (interquartile (IQ): 40.4–53.5). All patients received 45 Gy in 25 fractions associated with extended field radiotherapy in patients with stage IVB disease, and a with nodal boost in patients with nodal metastases. Then, all patients received a brachytherapy boost. OTT, including brachytherapy, was 47 days (IQ: 44–52 days). Median CTV_HR_ volume was 22.2 cm^3^ (range: 16.8–31.3 cm^3^). Brachytherapy was delivered in one fraction in 182 (87.1%) patients and in two fractions in 27 (12.9%) patients. Median D_90_CTV_HR_ was 81.3Gy_EQD2_ (95% CI: 74.5–89 Gy_EQD2_), median D_90_CTV_IR_ was 68.2 Gy_EQD2_ (65.7–71.5 Gy_EQD2_). Concurrent chemotherapy was cisplatin in 182 (87.1%) patients and carboplatin in 27 (12.9%) because of renal impairment. The number of cycles was 4 in 55 (19.1%) patients and 5 in 154 (73.7%). Characteristics of patients, tumors and treatments are detailed in Table 1.

### 3.2. Patterns of Relapse

Median follow-up was 5.5 years (range: 0.7–12.6 years) without a difference according to the number of cycles (*p* = 0.879). Sixty-six patients (31.6%) experienced tumor relapse: local in 24 (11.5%), regional (including para-aortic) in 37 (17.7%) and metastatic in 35 (16.8%). A total of 48 patients (23.0%) experienced locoregional failure (local and/or regional). More frequent relapses of all types (local, regional, distant metastatic) and deaths were seen among patients receiving four cycles (Table 2). There was no significant difference according to whether all cycles were delivered during EBRT or whether one of the cycles was delivered concomitantly with brachytherapy.

### 3.3. Survival and Disease Control Analysis

At the last follow-up, 51 patients had died (24.4%), all from disease. In the whole cohort, 3-year estimated OS was 79.9% (Standard Error (SE): 2.9%), PFS was 72.0% (SE: 3.2%), LFS was 77.7% (SE:2.9), RFS was 74.9% (SE: 3.1%), LRFS was 73.5% (SE: 3.1%) and MFS was 75.2% (SE: 3.0%). After three years, local control (LC) probability was 89.5% (SE: 2.2%), locoregional control (LRC) was 78.8% (SE: 2.9%) and distant metastatic control (DMC) was 75.2% (SE: 3.1%).

Patients receiving five chemotherapy cycles had a better outcome regarding all endpoints at three years, including LC rate (93.9% vs. 77.2%; Hazard Ratio (HR): 0.306; 95% CI: 0.137–0.681; *p* < 0.05) and DMC (69.1% vs. 90.5 %; HR = 0.280; 95% CI: 0.144–0.549) (Table 2).

### 3.4. Subgroup Analysis and Interaction with Brachytherapy Parameters

Significant and strong correlations were observed between the number of chemotherapy cycles and the following variables: FIGO stage (≤IIB or ≥IIB) and D_90_CTV_HR_ dose (≤80Gy_EQD2_ or >80Gy_EQD2_). Because of these correlations, multivariate analyses could not be done but subgroup analyses were performed to further examine the chemotherapy. In patients with a FIGO stage ≤IIB or with a CTV_HR_ > 25 cm^3^, receiving a fifth chemotherapy cycle was associated with a significantly better outcome for OS, PFS, LFS, RFS, LRFS, DMFS and for LC. There was no effect on survival or on disease control among patients with tumor stage ≥IIIA or those that had a CTV_HR_ ≤ 25 cm^3^ or those that had neutrophilia. LRFS was improved only in N0 patients (Table 3).

**Figure 1:**
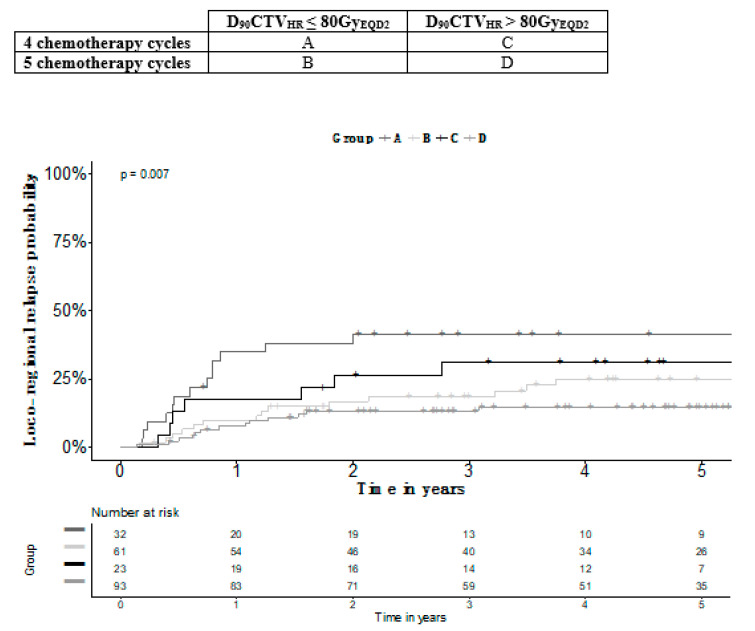
Relapse rate according to the number of chemotherapy cycles and D_90_CTV_HR_ (*n* = 209).

In patients with a D_90_CTV_HR_ ≤ 80Gy_EQD2_, an additional cycle was associated with a better outcome for all survival endpoints. When good coverage was achieved (D_90_CTV_HR_ > 80Gy_EQD2_), receiving five cycles was associated with better LFS, LRFS and PFS. Three-year survival rates according to the number of chemotherapy cycles and D_90_CTV_HR_ are shown in Table 4**.** Patients receiving four cycles and achieving D_90_CTV_HR_ > 80Gy_EQD2_ had the same LRC as patients receiving five cycles and achieving D_90_CTV_HR_ ≤ 80Gy_EQD2_ (*p* = 0.75) (Figure 1).

### 3.5. Propensity Score Matching.

**Figure 2:**
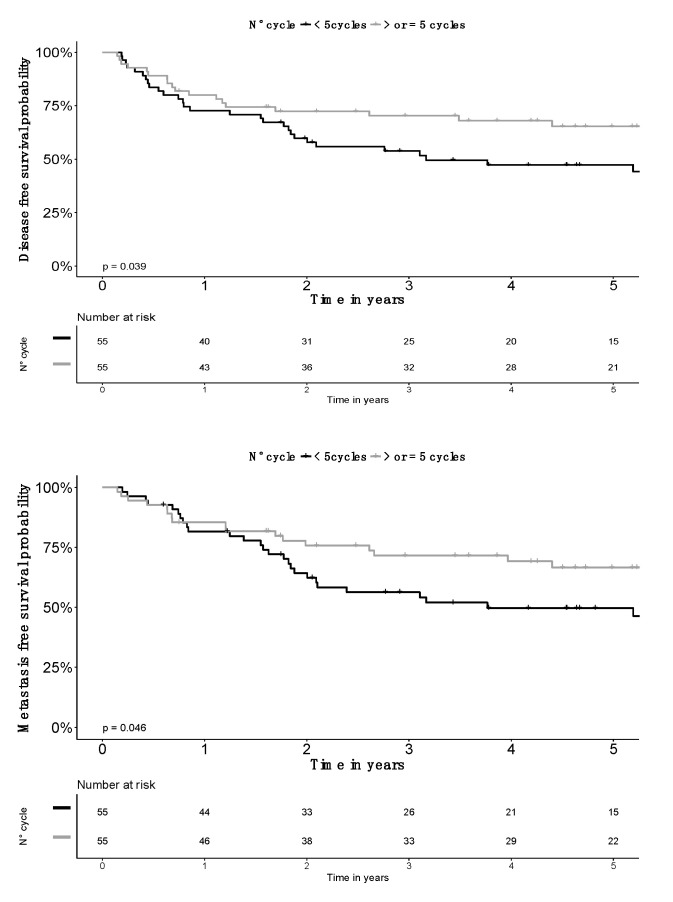
Distant metastatic failure-free and disease-free survival probability according to the number of chemotherapy cycles delivered after propensity score matching (*n* = 110). Grey line for > or = 5 cycles and black for <5 cycles.

**Figure 3:**
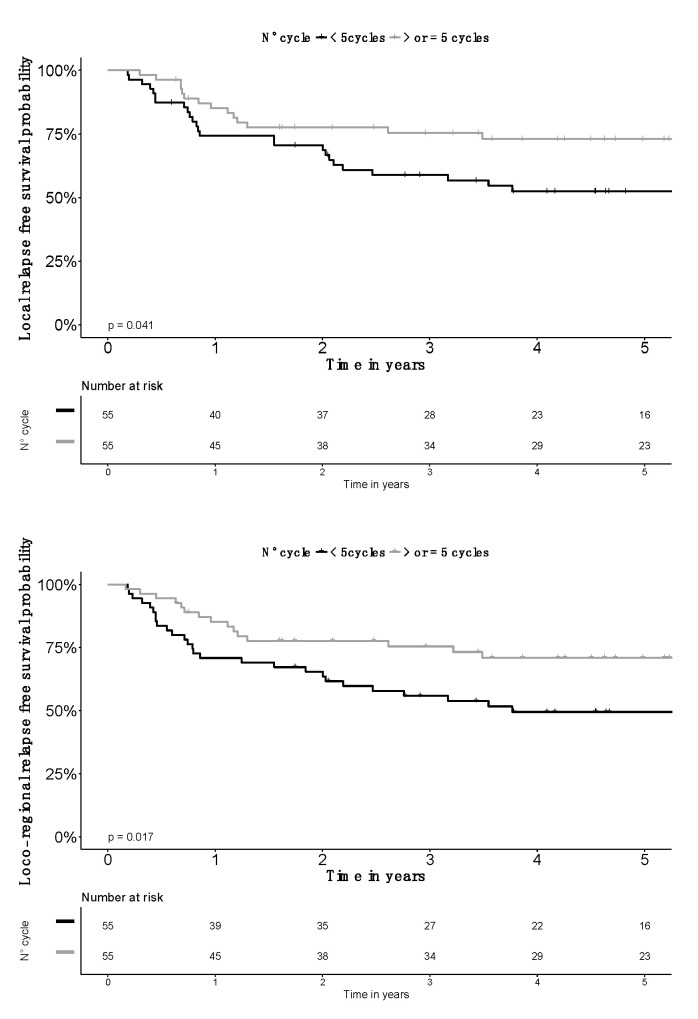
Local relapse-free and locoregional relapse-free survival probability according to number of chemotherapy cycles delivered after propensity score matching (PSM) on CTVHR volume, D90CTVHR and FIGO stage (*n* = 110). Grey line for > or = 5 cycles and black for <5 cycles.

Compared to patients receiving four cycles, those receiving five cycles had less advanced FIGO stages, smaller CTV_HR_ volumes and higher D_90_CTV_HR_ doses. Incidence of pelvic nodal metastases was similar. Neutrophil count was available in 175 patients, without a difference in neutrophila prevalence (neutrophils > 7500/mm^3^) according to study groups (Table 2). To minimize biases, a propensity score matching (PSM) analysis was done, balancing for FIGO stage, CTV_HR_ volume and D_90_CTV_HR_ as covariates, with a 1:1 ratio and an optimal matching resulting in all patients receiving four cycles being matched with a patient receiving five cycles. Groups were comparable for age, all tumor characteristics (including nodal status and neutrophils count), performance status and radiotherapy characteristics (Supplementary material Table S1). OS was not significantly improved, but a fifth cycle was an independent significant factor for other survival endpoints, including PFS, LFS, LRFS and DMFS (Figure 2 and Figure 3). In RMST analysis, the largest amount of life expectancy absolute gain was for LRFS: 1.5 years (95% CI: 0.3–2.7; *p* = 0.017) (Supplementary material Table S2). LRC rate was better at three years: 62.8% (SE: 6.6%) in patients receiving four cycles, vs. 82.9% (SE: 5.2%) in patients receiving five cycles (HR: 0.45; 95% CI: 0.212–0.970).

### 3.6. Radiation-Induced Toxicities

A fifth cycle did not increase in-field toxicities: all toxicities included, grade 2+ toxicities were reported in 77/154 (36.8%) patients receiving four cycles and 23/55 (41.8%) patients receiving five cycles (*p* = 0.346). Grade 3+ toxicities were reported in 2/55 (1.0%) vs. 9/154 (5.4%) (*p* = 0.731). After receiving four or five cycles, three-year estimated survival without Grade 2+ and Grade 3+ toxicity was 51% (SE: 4.4%) vs. 57.4% (SE: 7.5%) and was 100% vs. 96.4% (SE: 1.6%), respectively (not significant). No effect was observed when examining individual rectal, urinary, or vaginal toxicities (Table 2). There was no significant difference in terms of locoregional toxicities according to whether the fifth chemotherapy cycle had been delivered during EBRT or at the time of brachytherapy. Although hematological toxicity was not assessed due to lacking data, OTT was not increased by an additional chemotherapy cycle.

## 4. Discussion

The benefit of CCRT in patients treated for an LACC has been established from meta-analyses showing that concurrent chemotherapy reduced local and distant recurrences, mainly for patients with stage I-II tumors [1,2,3,4,5,6,7,8,16]. In 2017, a systematic review and meta-analysis examined the benefit of concurrent chemotherapy and confirmed that CCRT improved complete response rate (+10.2%, *p* = 0.027), LRC (+8.4%, *p* < 0.001) and OS (+7.5%, *p* < 0.001). The benefit of CCRT was at the expense of a higher incidence of severe acute toxicities, without an effect on late sequelae. No difference was found according to the use of cisplatin [17]. The target number of concurrent chemotherapy cycles in the era of IGABT is, however, uncertain.

It has been shown from retrospective data and a large prospective non-randomized study that delivering a modern treatment based on IGABT improved LC and disease-free survival [9,10,11,12,13,17]. Local relapse has become rare, and therefore it is uncertain whether CCRT may still improve local control in this context. A previous study has examined prognostic factors for local control in 225 patients treated for LACC with IGABT, including 95% treated with CCRT. In univariate analysis, chemotherapy use was not significant for LC probability [18]. This study, however, did not examine the number of cycles, and some patients were considered as having received chemotherapy although only suboptimal chemotherapy was delivered (one or two cycles), potentially because of poor general health status or tumor-related complications. The same limitations are found in most IGABT studies, which have focused on the brachytherapy dose effect, without thoroughly considering systemic treatments. In a multicenter retro-EMBRACE study, 731 patients received definitive EBRT followed by IGABT and 77.4% received concurrent chemotherapy. CCRT had a significant impact on three-year OS: 78% vs. 60% in the non-chemotherapy group (*p* ≤ 0.001). No impact on local control was reported. Again, no detail was provided on the number of cycles, and chemotherapy was not examined as an independent factor [19].

In an ongoing international study on MRI-guided brachytherapy in LACC (EMBRACE 2), chemotherapy has been recognized as an important part of treatment efficacy, and weekly concomitant cisplatin (40 mg/m^2^) is considered as the standard unless chemotherapy is precluded by patient age, co-morbidity or toxicity, and the treatment aim is to apply a minimum of five cycles [20]. This objective is based on a retrospective institutional series of IGABT suggesting that the total number of cycles received during the treatment would play an important role in systemic control of high-risk patients, defined as those with pelvic nodal metastases or FIGO stage >IIB. Schmid and colleagues have examined patterns of distant relapse in patients treated with CCRT and IGABT. In multivariate analysis, FIGO stage, lymph node status and the extent of tumor regression during treatment were predictive of distant metastases. The CTV_HR_ volume and D_90_CTV_HR_ were significant factors for distant metastasis in univariate analysis for high-risk patients, suggesting that an inability to deliver an optimal brachytherapy dose might reflect tumor bulk and be indicative of a higher risk of distant failure. The number of chemotherapy cycles was also significant [14]. Patients with local failures were, however, excluded, which introduces a bias to the analysis of chemotherapy effects [21].

To date, no published data has examined the benefit of chemotherapy on local control in patients treated with dose escalation. This study more thoroughly analyzed the benefit of concurrent chemotherapy in terms of LC in patients treated with IGABT. In our experience, all patients treated for an LACC are delivered CCRT apart in case of contraindication. In order to minimize biases that might impact on the delivery of an adequate chemotherapy regimen, we included only patients receiving four or five cycles to examine the effect of an additional cycle. The finding that patients receiving only four cycles had larger CTV_HR_, a lower D_90_CTV_HR_ and more advanced FIGO stages confirmed us a posteriori in this decision, and highlights the difficulty in appraising treatment effects in this clinical situation where numerous treatment parameters are dependent on each other.

In this homogenous series of patients, we found that patients receiving five cycles had a better outcome than those receiving four cycles. The effect was on metastatic events, which had been shown before [14], but also on local and locoregional controls, which had been shown in previous meta-analyses with outdated brachytherapy techniques but is a novel finding in the era of IGABT. In the subgroup analysis, our data are consistent with previous meta-analyses showing that the benefit of CCRT that is mainly in patients with FIGO stage ≤IIB may be due to the lower impact on distant disease relapse more commonly found in more advanced stages. We also found the lack of benefit of five cycles in patients with neutrophilia, which is a powerful independent poor prognostic factor associated with a high risk of metastases [22]. Although one cannot rule out the possibility of an insufficient statistical power to show an effect, these patients have a very poor prognosis in terms of metastatic risk, and these patients may warrant a more drastic systemic intensification.

A novel finding was that patients with the largest CTV_HR_ at the time of brachytherapy derived the greatest benefit of an additional cycle, suggesting that an intensification of systemic treatment may be part of the optimization process applied in patients who have a large residual disease, in addition to dose escalation [18]. PDR use, which requires having patients hospitalized for two to three days, seems particularly appropriate to fulfill the objective of achieving systemic intensification because it gives the opportunity to deliver an additional cycle. In our series, the fifth cycle was delivered at the time of brachytherapy in almost one half of patients. Although this study did not have the statistical power to identify a differential effect according to whether chemotherapy is delivered during EBRT or at the time of IGABT, our results suggest that an additional cycle should still be considered at the time of brachytherapy if the patient’s blood cell count is within a normal range. More thorough analyses are needed to investigate the true nature of interactions between radiation and chemotherapy, which might be additive as well as synergic [23].

In an attempt to further reduce the bias due to potential confounding covariates, a propensity score was done, matching groups for FIGO stage, CTV_HR_ volume and D_90_CTV_HR_ as covariates. With a qualitative control for potential biases, the propensity score matching confirmed that an additional cycle was an independent factor improving PFS, DMFS, LFS and LRFS, strongly suggesting that all efforts should be done to provide these patients with at least five chemotherapy cycles. The highest life expectancy increase was in terms of LRFS. Patients achieving poor CTV_HR_ coverage and receiving five cycles had the same outcome as patients achieving CTV_HR_ >80Gy_EQD2_ and receiving only four cycles, suggesting that CCRT might partially compensate for the inability to achieve dosimetric treatment planning aims through the least additive effects. Interestingly, even patients who achieved a D_90_CTV_HR_ >80Gy_EQD2_ had their LFS improved. This suggests that there is still an opportunity for systemic treatment research in order to improve LRC, even in the era of IGABT, through in-field cooperation. Of importance, we did not show any significant increase in long-term morbidity, which is in line with published data showing that CCRT increases acute toxicity, without any effect on long-term toxicity [16]. Delayed medullary effects were not assessed. However, long-term safety and an ability to keep OTT <55 days were not compromised by intensive systemic treatment.

This study has limitations inherent to its retrospective nature, and even a propensity analysis could not completely eradicate analyses and reporting bias. First, our cohort population is relatively young in age and with less-advanced tumors as compared to other IGABT series. The ability to deliver five cycles and the benefit of fulfilling this aim might be different in a cohort of patients more advanced in age or in tumor stages. Second, the D_90_CTV_HR_ reported here was slightly lower in this PDR cohort, as compared to other dosimetric data with high-dose rate IGABT [24]. Since then, we have increased our planning aim to achieve at least 85–90 Gy in 90% of patients by increasing the use of interstitial implants in order to increase local control probability. The benefit of chemotherapy might be slightly different if higher doses are applied to the CTV_HR_. Another limitation is that we included patients receiving carboplatin, which may be less effective than cisplatin, although this is not demonstrated. Finally, by excluding patients who received fewer than four cycles to minimize analysis biases, we could not conclude on the relative or absolute benefit of an additional chemotherapy cycle.

In spite of these limitations, our data suggest that delivering five chemotherapy cycles is better than delivering four cycles. These results are consistent with data published in head and neck cancers, showing that cisplatin dose intensity <200 mg/m² has a detrimental impact on OS [25,26]. In weekly cisplatin schedules, at least five cycles are required to achieve the same total dose of 200 mg/m². These data confirm the potential role of systemic agents in LACC in an attempt to improve LRC, provided that a minimal dose threshold is achieved. The ongoing OUTBACK study should provide meaningful information on the benefit of adjuvant chemotherapy in these patients to reduce the metastatic risk [27]. While it is likely that a randomized trial will never ask the question of the number of chemotherapy cycles, prospective results of EMBRACE studies are awaited to provide a higher level of evidence on this issue. Beyond the question of chemotherapy, the next steps of brachytherapy optimization should include pharmacological approaches to limit the risk of systemic failure [28].

## 5. Conclusions

This series provides the first clinical rationale that delivering five cycles compared to four cycles of concurrent chemotherapy may improve local control in patients treated for a LACC by CCRT plus IGABT. Increasing the total number of cycles from four to five was associated with a benefit in all survival endpoints. Local control was improved even in patients with an adequate CTV_HR_ coverage, suggesting that there remains a role for systemic interventions not only for decreasing distant failures, but also for in-field cooperative effects. Delivering an additional cycle at a time of PDR brachytherapy did not increase morbidity and permitted an increase in chemotherapy dose intensity.

## Figures and Tables

**Table 1:** Comparison of patients, tumors and treatment characteristics according to the number of chemotherapy cycles delivered (*n* = 209).

Characteristics	Number (%) or Median (IQ)		*p*-Value
	4 Cycles Delivered	5 Cycles Delivered	
Patients			
Number of patients	55	154	
Age (years)	47.0 (42.0–53.7)	47.4 (39.8–53.5)	0.690
Tobacco use	20/55 (36.4)	51/154 (33.1)	0.740
PS			
0	23/55 (41.9)	92/154 (59.7)	0.055
1	28/55 (50.9)	57/154 (37.0)	
2	4/55 (7.3)	5/154 (3.2)	
Tumors			
SCC	48/55 (87.3)	124/154 (80.5)	0.308
Poor differentiation	16/55 (29.1)	30/154 (19.5)	0.184
FIGO stage			
IB1	0/55 (0)	4/154 (2.6)	0.011 *
IB2	17/55 (30.9)	42/154 (27.3)	
IIA	0/55 (0)	11/154 (7.1)	
IIB	21/55 (38.2)	72/154 (46.8)	
IIIA	0/55 (0)	4/154 (2.6)	
IIIB	9/55 (16.4)	6/154 (3.9)	
IVA	3/55 (5.5)	3/154 (1.9)	
IVB	5/55 (9.1)	12/154 (7.8)	
Pelvic nodal metastases	23/55 (41.8)	60/154 (40)	0.640
Neutrophilia at diagnosis	13/43 (30.2)	29/132 (22.0)	0.306
Treatments			
Cycle during IGABT	21/55 (38.2)	80/154 (51.9)	0.086
OTT (days)	47 (43–52)	48 (44–52)	0.164
CTV_HR_ volume (cm^3^)	26.6 (18.5–40.9)	21.4 (16.3–28.1)	0.014 *
D_90_CTV_IR_ (Gy _EQD2_)	67.2 (60.8–70.0)	68.5 (66.2–72.0)	0.006 *
D_90_CTV_HR_ (Gy _EQD2_)	78.7 (74.0–86.1)	82.8 (74.9–90.4)	0.014 *
TRAK	1.80 (1.58–1.96)	1.74 (1.57–1.93)	0.328
2 fractions used	12/55 (21.1)	15/154 (9.7)	0.031 *

ADK: adenocarcinoma; CTV: clinical target volume; cm^3^: cubic centimeters; EBRT: external beam radiotherapy; Gy: Gray; HR: High Risk; IR: intermediate risk, EQD2: equivalent dose in 2Gy fraction; IGABT: image guided adaptive brachytherapy; IQ: interquartile 25–75; OTT: overall treatment time, including EBRT and IGABT; PAN: para-aortic node; PS: performance status; SCC: squamous cell carcinoma; *: significant comparison; FIGO: Fédération Internationale de Gynécologie Obstétrique.

**Table 2:** Patterns of relapse, toxicities and 3-year (3-y) survival according to the number of chemotherapy cycles delivered (*n* = 209).

**Number of Cycles Delivered**	**4 Cycles (*n* = 55)**	**5 Cycles (*n* = 154)**	***p*-Value**
Death (%)	24 (43.6)	27 (17.5)	0.000 *
Relapse (%)	28 (50.9)	38 (24.7)	0.000 *
Local relapse (%)	12 (21.8)	12 (7.8)	0.012 *
Regional relapse (%)	15 (27.3)	22 (14.3)	0.039 *
Loco-regionale relapse (%)	20 (36.4)	28 (18.2)	0.009 *
Metastasis relapse (%)	18 (32.7)	17 (11.0)	0.001 *
**Number of Cycles Delivered**	**4 Cycles**	**5 Cycles**	**HR (95% CI)**
3-y OS (SE)	62.0% (6.7)	87.8% (2.7)	0.340 (0.196–0.589) *
3-y PFS (SE)	53.4% (6.8)	78.8% (3.3)	0.388 (0.242–0.624) *
3-y LFS (SE)	58.9% (6.8)	84.4% (3.0)	0.345 (0.202–0.589) *
3-y RFS (SE)	59.2% (6.8)	80.5% (3.3)	0.405 (0.241–0.682) *
3-y LFRFS (SE)	55.9% (6.8)	80.0% (3.3)	0.389 (0.237–0.637) *
3-y DMFS (SE)	56.3% (6.9)	81.9% (3.2)	0.346 (0.208–0.573) *
3-y LC (SE)	77.2% (5.8)	93.9% (2.0)	0.306 (0.137–0.681) *
3-y LRC (SE)	62.8% (6.6)	84.6% (3.0)	0.429 (0.242–0.762) *
3-y DMC (SE)	69.1% (6.7)	90.5% (2.4)	0.280 (0.144–0.545) *
**Toxicity**	**4 Cycles**	**5 Cycles**	***p*-Value**
Grade 2+ late toxicities (%)	23 (41.8)	77 (50.0)	0.346
Grade 3+ late toxicities (%)	2 (3.6)	9 (5.8)	0.731

SE: standard error; OS: overall survival; PFS: progression free survival; LFS: local free survival; RFS: regional free survival; LRFS: loco-regional free survival; DMFS: distant metastasis free survival; LC: local control; RC: regional control; LRC: loco-regional control; LRC: loco-regional control; DMC: distant metastatic control; IQ: interquartile 25–75; CI: confidence interval; HR: hazard ratio for death/event; *: significant comparison.

**Table 3:** Subgroup analysis of the benefit of the fifth chemotherapy according to tumor stage, D90 CTV_HR,_ CTV_HR_ volume, pelvic nodal status and neutrophils count. Hazard ratio for death/event are given (95% CI confidence intervals) (*n* = 209).

	**FIGO < IIIA**	**FIGO ≥ IIIA**	**N0**	**N1**	**Neutrophils ≤ 7500/mm^3^**
OS	0.278 (0.142–0.546) *	0.704 (0.271–10.827)	0.373 (0.169–0.823)*	0.329 (0.152–0.710) *	0.285 (0.126–0.646) *
PFS	0.344 (0.19–0.617) *	0.682 (0.310–10.498)	0.383 (0.192–0.765)*	0.412 (0.215–0.791) *	0.415 (0.214–0.808)
LFS	0.319 (0.165–0.616) *	0.562 (0.223–10.417)	0.389 (0.184–0.824)*	0.307 (0.142–0.663) *	0.298 (0.133–0.660)
LRFS	0.343 (0.186–0.343) *	0.703 (0.303–10.630)	0.382 (0.190–0.768)*	0.408 (0.203–0.822)	0
DMFS	0.344 (0.180–0.655) *	0.542 (0.237–10.236)	0.327 (0.155–0.689)*	0.386 (0.194–0.770) *	0.369 (0.176–0.774)
LC	0.340 (0.127–0.914) *	0.346 (0.082–10.452)	0.340 (0.127–0.914)*	0.264 (0.080–0.865) *	0.301 (0.091–988)
LRC	0.439 (0.214–0.898) *	0.582 (0.218–10.554)	0.388 (0.170–0.886)*	0.475 (0.213–10.059)	0.482 (0.215–0.10.083)
DMC	0.286 (0.110–0.742) *	0.478 (0.187–10.223)	0.193 (0.069–0.545)*	0.401 (0.165–0.974)	
	**CTV_HR_ ≤ 25cm^3^**	**CTV_HR_ > 25cm^3^**	**D90 CTV_HR_ ≤ 80Gy_EQD2_**	**D90 CTV_HR_ > 80Gy_EQD2_**	**Neutrophils > 7500/mm^3^**
OS	0.569 (0.219–10.482)	0.304 (0.149–0.622) *	0.360 (0.179–0.723) *	0.399 (0.157–10.014)	0.464 (0.172–10.251)
PFS	0.598 (0.263–10.359)	0.369 (0.201–0.678) *	0.411 (0.222–0.761) *	0.431 (0.200–0.927) *	0.481 (0.193–0.1198)
LFS	0.482 (0.195–10.195)	0.339 (0.171–0.674) *	0.402 (0.205–0.789) *	0.341 (0.139–0.834) *	0.536 (0.203–10.412)
LRFS	0.664 (0.279–10.579)	0.346 (0.182–0.658) *	0.433 (0.231–0.813) *	0.415 (0.183–0.939) *	0.489 (0.193–10.242)
DMFS	0.583 (0.242–10.407)	0.310 (0.161–0.597) *	0.348 (0.183–0.662) *	0.437 (0.185–10.031)	0.442 (0.174–10.124)
LC	0.365 (0.103–10.295)	0.325 ( 0.112–0.938) *	0.379 (0.137–10.048)	0.289 (0.078–10.076)	0.769 (0.140–40.224)
LRC	0.611 (0.237–10.574)	0.406 (190–864) *	0.488 (0.232–10.028)	0.434 (0.173–10.088)	0.640 (0.209–10.959)
DMC	0.429 (0.143–10.280)	0.260 (0.107–0.634) *	0.303 (0.132–0.697) *	0.330 (0.105–10.040)	0.302 (0.091–10.128)

SE: standard error; OS: overall survival; PFS: progression free survival; LFS: local free survival; RFS: regional free survival; LRFS: loco-regional free survival; DMFS: distant metastasis free survival; DC: disease control; LC: local control; RC: regional control; LRC: loco-regional control; LRC: loco-regional control; DMC: distant metastatic control; *: significant values.

**Table 4:** Three-year survival depending D_90_CTV_HR_ and number of chemotherapy cycles (*n* = 209).

Number of Cycles	4	5	4	5	Overall *p*-Value	B versus C *p*-Value
D90CTVHR	≤80Gy_EQD2_	≤80Gy_EQD2_	>80Gy_EQD2_	>80 Gy_EQD2_		
Number of Patients	32	61	23	93		
OS (SE)	54.1% (9.1)	83.7% (4.7)	68.5% (9.9)	89.2% (3.4)	0.000	0.408
PFS (SE)	48.7% (9.0)	72.3% (5.6)	60.1% (10.4)	83.1% (4.0)	0.000	0.609
LRFS (SE)	49.6% (8.9)	72.3% (5.7)	64.7% (10.1)	85.4% (3.7)	0.000	0.711
LRC (SE)	58.6% (8.8)	81.4 (5.2)	69.7% (9.8)	86.7% (3.5)	0.007	0.752

E: standard error; OS: overall survival; PFS: progression free survival; LRFS: loco-regional free survival; DC: disease control; LRC: loco-regional control.

## Supplementary Materials

The following are available online at https://www.mdpi.com/2077-0383/9/6/1653/s1, Table S1: Characteristics of patients after propensity score matching on FIGO stage, CTVHR volume and D90CTVHR dose, Table S2: Restricted mean survival time analysis estimating the life expectancy benefit afforded by an additional cycle after propensity score matching (*n* = 110).
