# Supplementary material for: Interaction between the Number of Chemotherapy Cycles and Brachytherapy Dose/Volume Parameters in Locally Advanced Cervical Cancer Patients
Source: J Clin Med. 2020 Jun 1;9(6):1653. doi: 10.3390/jcm9061653 (PMC7356737; doi:10.3390/jcm9061653)
Supplement: Supplementary file 1 [file jcm-09-01653-s001.pdf]

Table S1. Characteristics of patients after propensity score matching on FIGO stage, CTV_{HR} volume, and $D_{90}CTV_{HR}$ dose.

**Covariates**		**4 Cycles Delivered**	**5 Cycles Delivered**	***p*-Value**
Patients				
Age (years)		47.0 (42.0–53.7)	48.0 (40.5–55.0)	0.632
Tobacco use		20/55 (36.4)	20/55 (36.4)	1.000
PS	0	23/55 (41.8)	32/55 (58.2)	0.229
	1	28/55 (50.9)	20/55 (41.7)	
	2	4/55 (7.3)	3/55 (5.5)	
Tumors				
SCC		48/55 (87.3)	46/55 (83.6)	0.788
Poor differentiation		16/55 (29.1)	14/55 (25.5)	0.831
FIGO stage	IB1	0/55 (0.0)	0/55 (0.0)	0.197
	IB2	17/55 (30.9)	12/55 (21.8)	
	IIA	0/55 (0)	2/55 (3.6)	
	IIB	21/55 (38.2)	26/55 (47.3)	
	IIIA	0/55 (0)	3/55 (5.5)	
	IIIB	9/55 (16.4)	4/55 (7.3)	
	IVA	3/55 (5.5)	2/55 (3.6)	
	IVB	5/55 (9.1)	6/55 (10.9)	
Pelvic nodal metastases		23/55 (41.8)	22/55 (40)	1.000
Neutrophilia at diagnosis		13/43 (30.2)	12/42 (28.6)	1.000
Treatments				
Overall treatment time (days)		47 (43–52)	47 (45–51)	0.441
Cycle during IGABT		21/55 (38.2)	29/55 (52.7)	0.180
CTV_{HR} volume (cm^3)		26.6 (18.5–40.9)	21.4 (16.3–28.1)	0.503
$D_{90}CTV_{IR}$ (Gy_{EQD2})		67.2 (60.8–70.0)	68.3 (64.1–70.8)	0.279
$D_{90}CTV_{HR}$ (Gy_{EQD2})		78.7 (74.0–86.1)	78.8 (72.1–87.7)	0.874
TRAK		1.80 (1.58–1.96)	1.77 (1.52–2.01)	0.917

FIGO: Fédération Internationale de Gynécologie Obstétrique; CTV_{HR}: high risk clinical target volume; TRAK : Total Reference Air Kerma; PS: performance status.

Table S2. Restricted mean survival time analysis estimating the life expectancy benefit afforded by an additional cycle after propensity score matching (*n* = 110).

Propensity Score Matching (*n* = 110)			
**Endpoint**	**Gain in Years**	**95%CI**	***p*-Value**
OS	1.1	-0.0–2.3	0.052
PFS	1.4	0.1–2.7	0.034*
LFS	1.3	0.1–2.5	0.038*
RFS	1.4	0.2–2.6	0.021*
LRFS	1.5	0.3–2.7	0.017*
MFS	1.3	0.1–2.5	0.039*
LC	0.5	-0.5–1.6	0.323
LRC	1.3	0.1–2.5	0.034*
DMC	1.1	-0.1–2.3	0.064

OS: overall survival; PFS: progression-free survival: LFS: local failure-free survival; RFS: regional failure-free survival; LRFS: locoregional failure-free survival; DMFS: distant metastasis failure-free survival; DMC: distant metastatic control; LC: local control; LRC: locoregional control; *significant comparison; CI: confidence interval.
